# Development of a New Negative Obstacle Sensor for Augmented Electric Wheelchair

**DOI:** 10.3390/s21196341

**Published:** 2021-09-23

**Authors:** Clément Favey, René Farcy, Julien Donnez, Jose Villanueva, Aziz Zogaghi

**Affiliations:** 1 Chaire Handicap & Technologie Polytech Paris-Saclay, Université Paris-Saclay, CEDEX, 91405 Orsay, France; 2 Laboratoire Aimé Cotton, Université Paris-Saclay, CNRS, CEDEX, 91405 Orsay, France; rene.farcy@universite-paris-saclay.fr (R.F.); julien.donnez@universite-paris-saclay.fr (J.D.); villanueva.jos@gmail.com (J.V.); aziz.zogaghi@universite-paris-saclay.fr (A.Z.)

**Keywords:** negative obstacles, detection, sensor, telemetry, laser, triangulation, disability, augmented wheelchair, electric wheelchair

## Abstract

Due to pathologies or age-related problems, in some disabled people, motor impairment is associated with cognitive and/or visual impairments. This combination of limitations unfortunately leads to an inability to move around independently. Indeed, their situation does not allow them to use a conventional electric wheelchair, for safety reasons, and for the moment there is no other technological solution providing safe movement capacity. This lack of access to an autonomous travel solution has the consequence of weakening the intellectual, personal, social, cultural and moral development, as well as the life expectancy, of the people concerned. In this context, our team is working on the development of an optoelectronic system that secures the displacement of electric wheelchairs. This is a large project that requires the development of several functionalities such as: the anti-collision of the wheelchair with its environment, the prevention of falls from the wheelchair on uneven levels, and the adaptation of the system mechanically and electronically to the majority of commercially available electric wheelchair models, among others. In this article, we introduce our solution for detecting dangerous height differences, also called “negative obstacles”, through the creation of a dedicated sensor. This sensor works by optical triangulation and can embed several laser beams in order to extend its detection zone. It has the particularity of being robust in direct sunlight and rain and has a sufficiently high measurement rate to be suitable for the displacement of electric wheelchairs. We develop an adapted algorithm, and point out compromises, in particular between the orientation of the laser beams and the maximal speed of the wheelchair.

## 1. Introduction

Whether it is due to age-related problems, a congenital disability or an accident, some people possess a combination of impairments that do not allow them to move independently. This combination is often of a motor nature, in addition to impaired cognition and/or visual field. For the time being, there is no technical solution to meet their needs in order to allow them to travel in complete safety.

They therefore find themselves in a passive displacement situation and require assistance from a caregiver or a family member.

Among the existing technical solutions, the electric wheelchair is the system that most closely matches the needs of this type of population in order to be able to move around; however, they have not been completely adapted, mainly due to safety problems. Indeed, an electric wheelchair weighs about 150 kg for an adult, so any failure in control by the driver can be very dangerous for the user as well as for surrounding persons and their environment, as in the case of collision, falls on staircases or tilting from a sidewalk, for example. A representative case for this type of situation (although not exclusive) is people with cerebral palsy. This pathology is likely to cause uncontrolled movements, a limitation of gestures, and visual impairment or cognitive problems for the people concerned. The expression of this pathology is specific to each person, with respect to both the nature of the impairments encountered and their severity.

- With regard to motor impairments, these mostly comprise spasticity, dystonia, ataxia, tremors, hemiplegia or hemiparesis.
- Visual impairments are generally a limitation of the field of vision coupled with poor acuity, which in the worst cases results in near blindness.
- Cognitive impairments mainly concern the capacity for learning, planning, spatial and temporal representation, management of emotions, etc.

All these impairments can, independently or in combination, compromise the control of the wheelchair. This is why, for those most affected, it is not possible to drive an electric wheelchair alone in complete safety.

Today there is a medical consensus on the benefits of the ability to move independently and the harm that immobilism can cause, both for the youngest [1] and the oldest [2,3]. In summary, there is a strong relation between autonomous displacement and the intellectual, personal, social and cultural development of any person, with or without disability. Moreover, the ability to move has an impact on life expectancy [4]. Then, there is the need to find reliable and robust solutions to include greater access to autonomous mobility.

Research on the development of new solutions allowing access to autonomous travel began in the early 1990s. The vast majority of these projects attempted to add an anti-collision function to electric wheelchairs, in order to allow navigation without hindrance. In this context, various technical solutions, and in particular sensors, have been investigated. Most of these are ultrasound sonars that are placed around the perimeter of the chair in order to detect the environment close to the chair. The best known ultrasound sonars are those used in the NavChair [5,6], Feego [7] and SYSIASS projects [8,9]. Some projects, such as UMIDAM [10], RobChair [11] and SYSIASS have also added infrared sensors to complete the shortcomings of ultrasound sensors. New sensors have recently emerged. Among them we can mention LiDAR, single fixed beam, multiple fixed beams or rotating lasers in order to detect obstacles and determine the profile of the environment of the electric wheelchair, notably in the work of Grewal [12], Arnay [13] or the SmartWheelchair project [14] and the iChair project [15,16]. Özcelikörs [17] and Mittal [18] used the Kinect camera system in order to obtain a 3D map of the frontal obstacles, as well as their size and position. Other approaches use video processing, whether in mono- or stereovision, in order to detect particular objects or dangerous situations [19,20].

Very few works have been devoted to preventing falls and the tipping of chairs from sidewalks onto roadways or down stairs. Two approaches have been investigated: those using a mechanical system and those using an electromechanical system that prevents the chair from tilting and therefore falling on its side on any terrain [21]. Although this avoids the possible damage associated with a fall of the wheelchair, this type of system does not intervene in the control of the wheelchair, and therefore does not prevent the latter from ending up on the road, in the middle of traffic, in the event of tilting off a sidewalk. The chair will remain upright, but on the road. Then there are those works that seek to detect dangerous unevenness in order to prevent the wheelchair from moving there, like the work of Devigne [22] and Mazo [10], who are both interested in developing a system based on infrared sensors, in order to detect differences in height within the framework of sidewalks or stairs. We also broadened our research to negative obstacle detection systems not applied specifically to electric wheelchairs, for example through the work of Karunasekera [23], who used a stereovision system, or that of Larson and Trivedi, which was based on a 3D LiDAR mounted on the roof of a car [24], which seem to be efficient systems, but not well suited to the circumstances of daily use of an electric wheelchair (e.g., with respect to the size of the sensors, the energy required, or the computing power required).

## 2. Materials and Methods

In this work we introduce the sensors and methods developed by our group. Our aim was to add an anti-fall function to an electric wheelchair based on detection of height differences (sidewalks, stairs, etc.). It is worth noting that anti-collision functions are also being developed in our research program, but they are not within the scope of the present work.

### 2.1. Generalized Wheel Protection

Although falls from sidewalks are the most frequent accidents, the system still has to correctly apprehend all situations of unevenness. Indeed, even if, in specialized centers, the stairs are protected by barriers, for example, we are ultimately working from a perspective assuming daily use, indoors and outdoors, in centers, at home, or in public places. Thus, the function to be fulfilled by the system can be extended to generalized wheel protection. Indeed, for someone to be in a tipping situation, one or more wheels must necessarily come off the road and end up in the void, subsequently causing the chair to fall. The system must therefore prevent the wheels from finding themselves in this situation by anticipating the presence of unevenness and by prohibiting this direction for the wheelchair. The detection system has to “see” the contours of the trajectory in advance, and must therefore be directed in front of the wheelchair wheels.

The complexity of this approach lies, on the one hand, in the diversity of types of potential height difference. While sidewalks and stairs are the most common dangers encountered, there are also a multitude of specific elements linked to the nature of the terrain on which the wheelchair moves (potholes, faults in the sidewalk, work areas, steep slopes, etc.). On the other hand, these danger zones can be located both directly in the path of the wheelchair, but also to its sides, so the detectors will have to monitor both the area in front of the wheels, as well as their surroundings.

Finally, the wheelchair must still be capable of crossing certain height differences, both positive and negative, without danger, as long as their sizes remain relatively small. This is the case with the lower parts of sidewalks, for example, for which the system must allow both ascent and descent. Our detection tool must therefore have sufficient precision to distinguish the limit between authorized height differences and dangerous ones.

### 2.2. Technology

Before choosing to develop our own sensor for this application, we naturally looked at existing solutions and projects in order to find a sensor that was suitable as it is. Unfortunately, this search was not successful. Below is a summary of the existing technologies mentioned in the introduction and the reasons to proceed differently.

Ultrasonic sonars have several drawbacks, including low angular resolution that does not allow precise measurements over 2 m. They cannot detect smooth surfaces at angles with incidences greater than 35 degrees because of the total reflection of the wave, and therefore the ultrasonic signal does not return towards the receiver located near the emitter. This reflection phenomenon also generates a high false-positive rate due to the multiple bounces of ultrasonic waves off smooth surfaces generating feedback from obstacles that are not in the direction of emission. In addition, they do not function properly in very humid weather or in the rain for a range of more than one meter due to the humidity shielding effect of ultrasonic waves.

Infrared sensors such as those in the Sharp GP2Y series are unusable in direct sunlight, thereby preventing outdoor use, as well as indoor use near windows in sunny weather.

The 3D camera makes it possible to construct a 3D image from a grid of infrared light beams projected into space by analyzing the backscattered rays. However, like conventional infrared sensors, 3D cameras are very sensitive to sunlight and are ineffective outdoors.

Image processing via video stream is generally used to highlight points of interest in the environment on a screen facing the user of the chair, or to recognize predefined objects. It is not used to measure the distance from surrounding obstacles because it is too weak for this purpose and requires too much memory, computing power and energy. In addition, it performs poorly in low-light conditions.

Rotating LiDARs can be used on the one hand to dynamically generate a 2D map of chair environment, and on the other hand to detect obstacles in the vicinity. However, the rotating devices present the following different drawbacks:

- The concealment of the scanning of the beam by the wheelchair or the user prevents monitoring of close frontal and lateral areas, mechanically creating large blind spot areas that will therefore not be protected,
- The permanent rotation of the LiDAR induces a reduced measurement rate at one point on the ground, which for a moving chair can result in accidents due to undetected dangers on the ground,
- Rotating LiDARs immune to direct sunlight are expensive, cumbersome and require a large amount of energy.

In addition, owing to their punctual nature, non-rotating LiDARs alone leave too many unmonitored areas in front of or at angles to the wheelchair, requiring a multiplicity of LiDARs to properly protect the wheelchair from the surrounding unevenness, which represents an energy and size constraint.

As stated previously, we therefore chose to build a custom sensor. Our goal was to combine the qualities of laser detection: energy concentrated at a point allowing a good signal-to-noise ratio (even under direct sunlight), satisfactory measurement accuracy, and low energy consumption (thanks to laser diodes), while bypassing its shortcomings, namely its punctual nature, which does not allow surface detection.

For this we developed a three-beam laser triangulation rangefinder. The choice of this optical triangulation rangefinder rather than time of flight is explained here by our desire to obtain a very compact sensor and a sub-centimetric resolution between 1 and 2 m, which is more difficult with time of flight.

### 2.3. Technical Characteristics

#### 2.3.1. Basic Scheme of the Sensor

**Figure 1:**
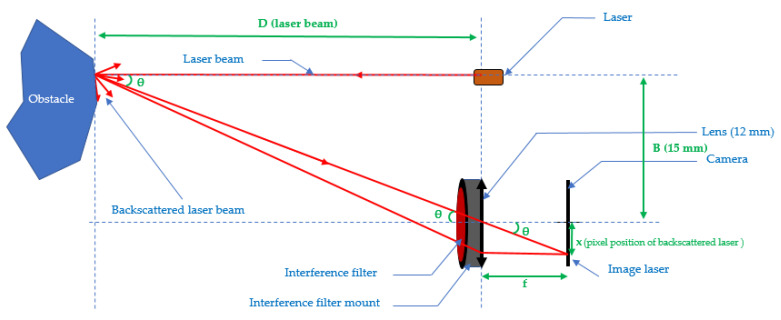
Presentation of basic scheme for one beam.

The triangulating laser telemeter is made of continuous laser diodes and a CMOS camera with a fixed distance B called *the base* between the optical axis of the camera and the laser beam. The basic setup is presented for only one beam in Figure 1 for simplicity.

Let B be the distance between the diodes of the CMOS matrix (also called the camera). The lens is placed at a distance f from the camera (corresponding to the focal length of the lens). We denote by *x* the position of the pixel of the matrix, multiplied by the size of a pixel (1.4 μm), on which the beam is backscattered. To determine the distance D between the sensor and the first obstacle encountered, we proceed as follows:

(1) $$\tan\theta =\frac{B}{D}=\frac{x}{f}$$

(2) $$D=\frac{B\;f}{x}$$

We have *B* = 15 mm and *f* = 12 mm. These values are fixed during the construction of the sensor. Measuring *x* then yields *D* at any given time. We use a continuous 650-nm laser diode and a 10-nm bandpass interference filter.

#### 2.3.2. Three-Beam Scheme of the Sensor

**Figure 2:**
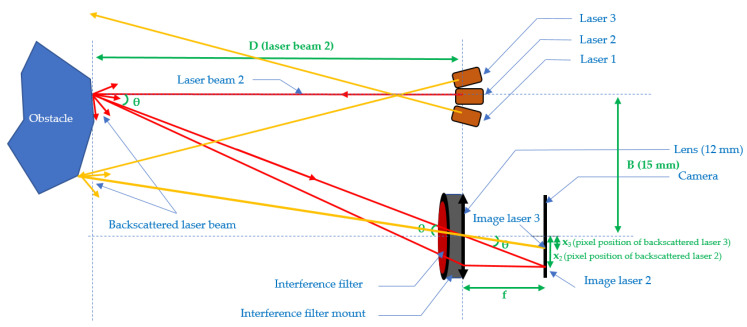
Basic scheme of the setup with three laser beams.

Figure 2 presents the three-beam telemeter setup. In the particular case of Figure 2, there are only two points on the CMOS camera, because only two beams come into contact with the obstacle.

#### 2.3.3. Practical Setup

**Figure 3:**
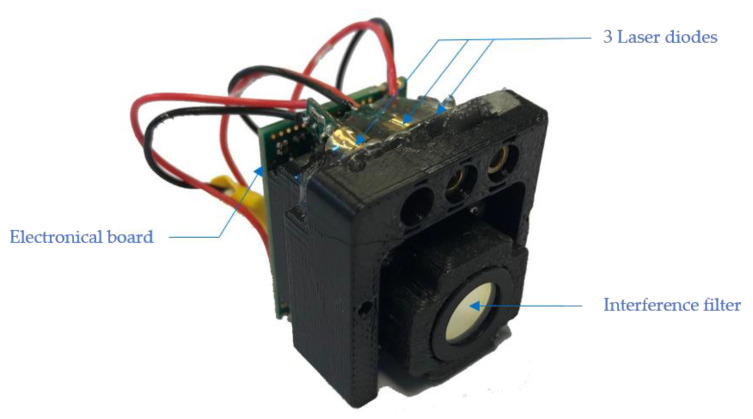
Presentation of the setup.

In Figure 3, the realization of the setup is presented. The used CMOS matrix is the OV2722 from Omnivision at a rate of 90 frames/s. The microcontroller used is the STM32F429 from ST Microelectronics. We use direct RAM access to analyze a window on the camera in real time.

#### 2.3.4. Resolution of the Telemeter

**Figure 4:**
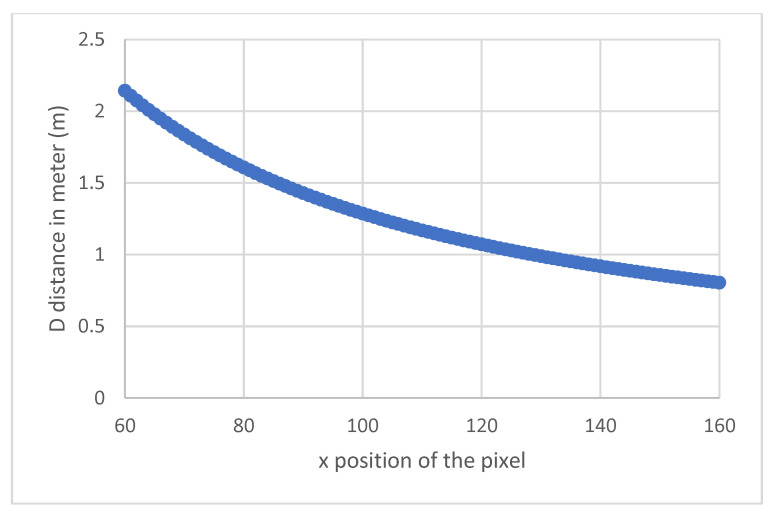
Variation of D with x for the central beam.

Figure 4 shows the variation of the measured distance D with the position of the pixel on which the beams return. These results are plotted only for the interval from 2.2 m to 0.80 m for the measured distances. In fact, obtaining information on the flatness of the ground, does not need to consider distances outside this interval, since the average distance measured for a flat ground is around 1.20 m. Restricting the interval thus makes it possible to obtain a better resolution.

**Figure 5:**
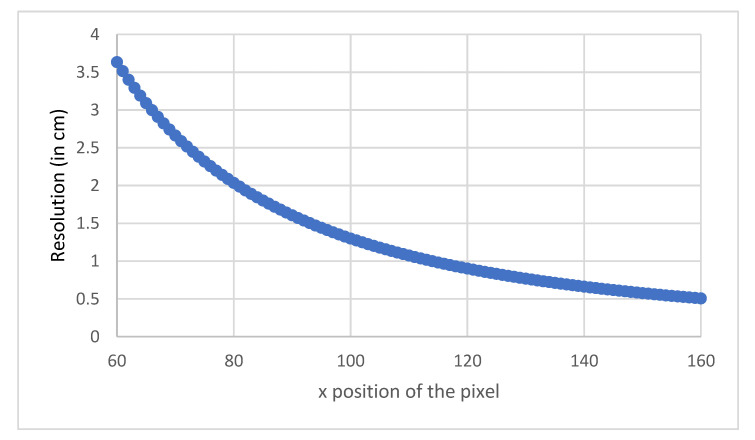
Variation of the resolution δD with x.

Figure 5 shows the resolution in the above interval as a function of the x position on which the laser beams are backscattered. As above, the distances range in the interval 0.8–2.2 m. We can see that the resolution evolves slightly according to the measured distance. The shorter the distance, the better the resolution. In our distance ranges, this is on average 1.5 cm per pixel, which is highly satisfactory for our purposes, allowing us to distinguish a sidewalk and a staircase, for example.

#### 2.3.5. Arrangement

**Figure 6:**
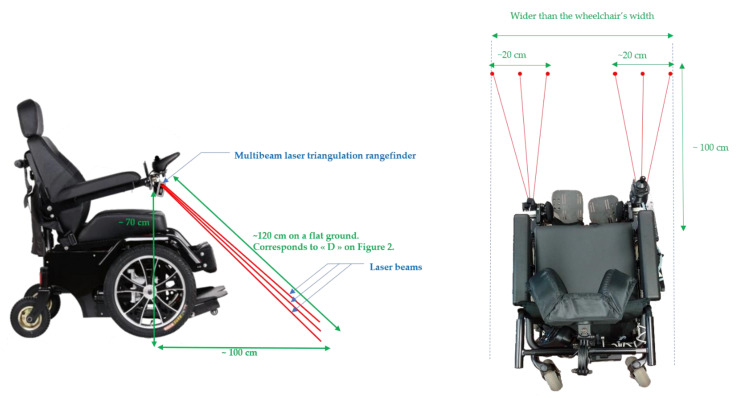
Sensor positions.

The setup below (Figure 6) must fulfill the following conditions:

- Concerning the anticipation distance in front of the wheelchair, two criteria must be satisfied. The first one is the necessary braking distance according to the speed of the wheelchair. The second one is the average size of the drop height likely to cause a fall from the wheelchair.
- The braking distance depends on several parameters: the speed of the wheelchair, the weight of the wheelchair and that of the user, the grip of the wheels and the nature of the terrain (slope, granularity, etc.). To obtain an order of magnitude of these distances, we carried out measurements for each speed level, on flat ground with a user with a weight of 70 kg (each measurement was carried out 5 times and then averaged).

We obtain the results summarized in Table 1.

**Figure 7:**
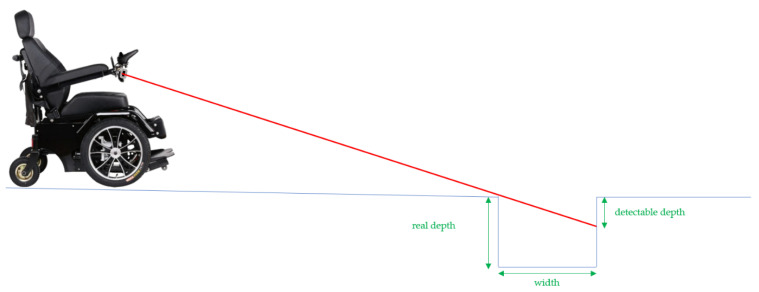
Detectable depth with beams pointing away.

The speeds in Table 1 correspond to maximal speeds 1 to 5 of a standard joystick of a Salsa M2 Sunrise Medical chair. Therefore, by interpolation, an anticipation distance of 100 cm ensures the protection of the wheelchair as long as it does not exceed approximately 5 km/h. For safety reasons, we limit ourselves to 3 km/h, in order to preserve a margin of error. To make this method usable at higher speeds, the beams would have to be raised so that they point farther away, anticipating any drop in height (see Figure 7). However, this causes a new problem, in particular due to the fact that the more the beam becomes grazing, the more capacity is lost to measure the real depth of obstacles on the ground.

Figure 7 shows that the system will not be able to measure the entire depth of the hollow and potentially authorizes the crossing of such an obstacle, without correctly assessing its dangerousness. By considering this effect, correlated with the necessary braking distance previously explained, we chose the positioning of our sensors. This is a reasonable compromise that allows us to measure hollows up to a depth of 14 cm and a width of 20 cm.

**Figure 8:**
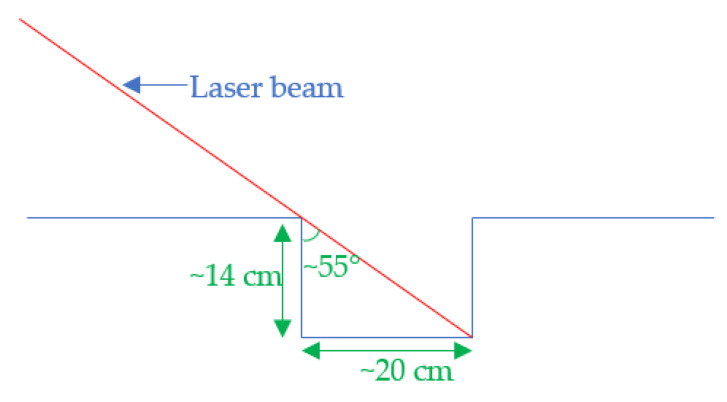
Limit of detection according to our disposition.

The 14 cm depth represents the maximum height that a sidewalk can reach. In this manner, even in the case of hollows deeper than 14 cm (and therefore of unknown real depth), the system will block the advance of the chair no matter what. The value of 20 cm indicated in Figure 8 is roughly the width of a hole a wheelchair would be able to move through due to the diameter of the wheels.

## 3. Results

### 3.1. Signals Obtained

Now we focus on the signals measured by our camera. It is worth mentioning that this is an RGB camera, in which the four pixels (B, G, R, G) are summed in order to obtain the information. Each pixel represents the sum of four pixels B, G, R, G side by side. For the sake of clarity, only one beam is shown.

**Figure 9:**
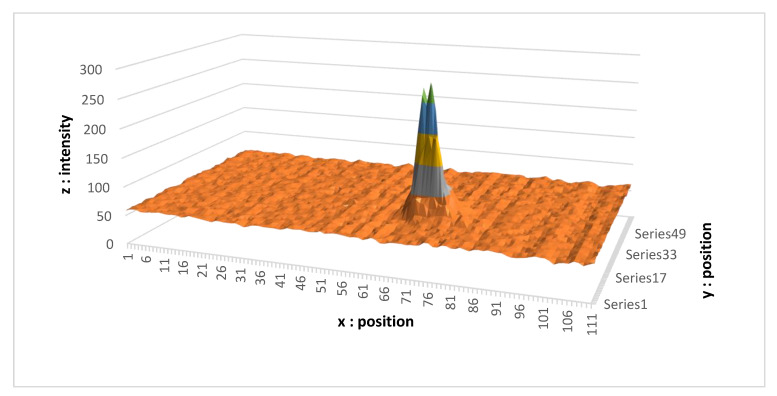
Signal obtained in poor luminosity.

Figure 9 was obtained in almost ideal conditions, i.e., the measurement of the distance was performed from a point on clear ground (with a good albedo), indoors (with very little stray light). We then obtained an easily identifiable Gaussian shape, where the maximum intensity of the peak is five times larger than that of the ambient light. The *z*-axis representing the intensity is unitless (it corresponds to the result of the analog-to-digital conversion). The *x* and *y* values represent the position of the pixel on the camera.

**Figure 10:**
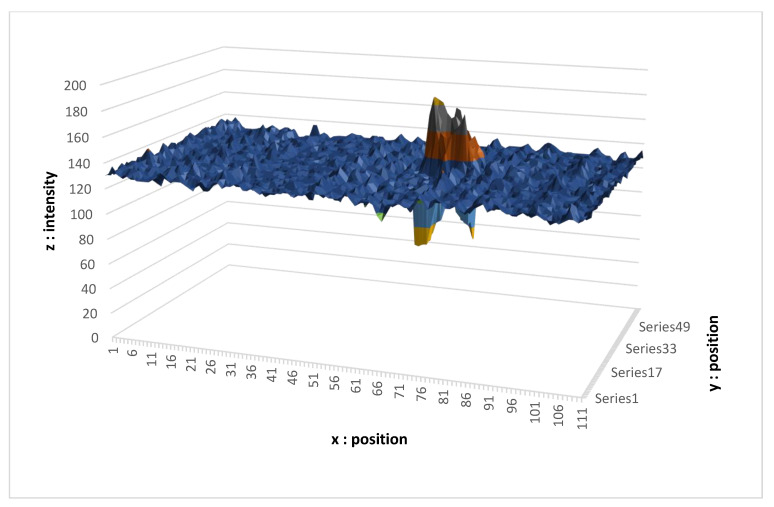
Signal obtained in very high luminosity.

The results shown in Figure 10 were obtained in difficult conditions, namely the measurement of the distance from a point on dark ground (lower albedo), outdoors in full sun (a lot of noise). Here, the maximum intensity of the peak is only 23% greater than the ambient luminosity. In addition, the shape of the spot is more flared, making the detection more complex. However, we can see that the dynamic of the sensor is large enough to obtain the distance information given by the position of the pike.

### 3.2. Algorithms

#### 3.2.1. Determination of Peak

Our algorithm detects the center of the peak and takes into account the difference in intensity between the brightest point on the camera and the others, as well as the shape of the zone. It is then necessary to measure the alternation of the derivatives of the measured values. Then, according to the sequence (positive–negative–positive), as well as the difference between the maximum value at the beginning of the negative derivative and the rest of the values of the matrix, it is possible to locate a peak. If the peak corresponds to a predefined shape that is 5 and 15 pixels in diameter, then we can confirm the presence or absence of the laser spot on the camera.

#### 3.2.2. Obtained Curves and Detection of Dangerous Height Differences

Following the presentation of the method of determination of the position of the backscattered laser spot, we focus on the evolution of its position as a function of time and on the progress of the electric wheelchair. It is worth stressing that each laser point is measured with a rate of 30 Hz (90 Hz/3, since each of the three laser diodes is turned on and off alternatively). A set of curves representing the evolution of the position of the measured points in relevant situations is shown below. For the sake of clarity, we present the values of only one of the three laser points.

Since our measurements are based on the position of the pixels to which our backscattered beams return, it should be noted that certain mechanical uncertainties apply to our measurements. The sensor is attached to the armrest of the electric wheelchair. No mechanical fixing is perfect, and its position varies regularly by a few millimeters. Additionally, the optical alignment between the lens and the camera card, by dint of being subjected to multiple vibrations (mainly due to the movement of the chair or to sudden shocks during its use), moves slightly on a regular basis. Thus, the value measured by the sensor at start-up, namely the distance between it and the flat ground, varies from one moment to another. For this reason, it is necessary to systematically start the system when it is on flat ground and to record this value. It is the difference from this base value that allows us to determine the presence or absence of dangerous unevenness.

Note that even when moving only on flat ground, the measured values will never be constant. They will oscillate slightly around an average value, which represents the distance between the sensor and the ground, but which will have a certain standard deviation due to the vibrations caused by the moving wheelchair.

The two graphs below highlight a typical problem to be solved in the system: how to distinguish a brutal drop from a gentle drop.

**Figure 11:**
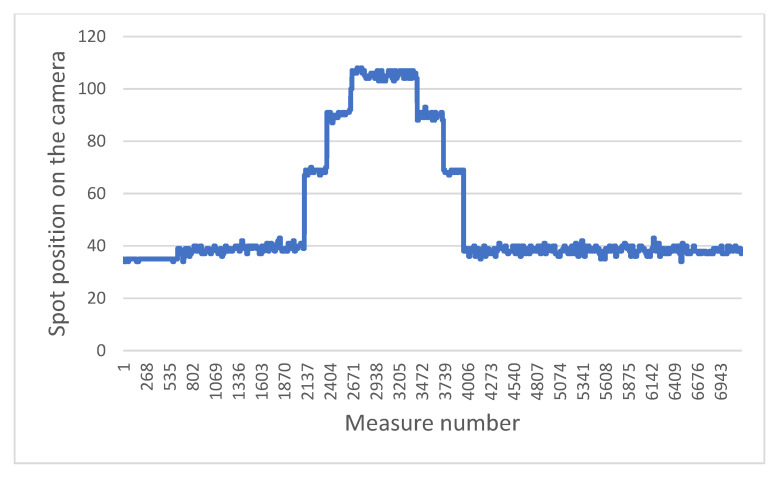
Evolution of position measured while the wheelchair moves toward stairs then goes backwards.

**Figure 12:**
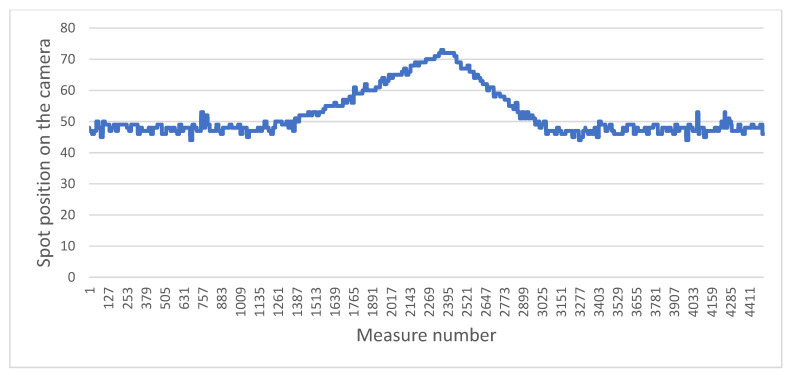
Evolution of position measured while the wheelchair moves toward a downward slope and then on flat ground.

Figure 11 presents the sensor measurements when the wheelchair moves towards and then backwards away from a staircase. Figure 12 presents the results obtained when the chair moves towards a gentle slope and then continues on flat ground. In both cases, when the laser beams fall into the drop, the values increase, as the ground “moves away”. The challenge is therefore to determine whether this is a dangerous situation or not. For that we focus on the value of the derivative of the distances. It can be easily observed that in the case of a staircase or in the same way for a sidewalk, the values increase sharply, compared to the case of a gentle slope. In the current situation, the wheelchair does not have an odometer.

To determine this derivative, a good compromise between the robustness, precision and speed of the measurement can be obtained by looking at the values of the last six measurements, then calculating the difference between the average of the last three points with the average of the first three. If this value exceeds a certain threshold, previously defined according to a series of tests, then it will be considered to be a dangerous difference in height. This calculation is performed in a sliding manner, i.e., each new value replaces the last of the six points and the calculation is carried out again over and over (with the five old points and the new one). In this way, only three new points in a vertical drop are enough to determine whether it is dangerous or not, which at our measurement rate allows a reaction time of 100 ms. This is very acceptable in light of the previously established fact that the wheelchair does not exceed a speed of 3 km/h, and therefore has nearly one meter to stop.

**Figure 13:**
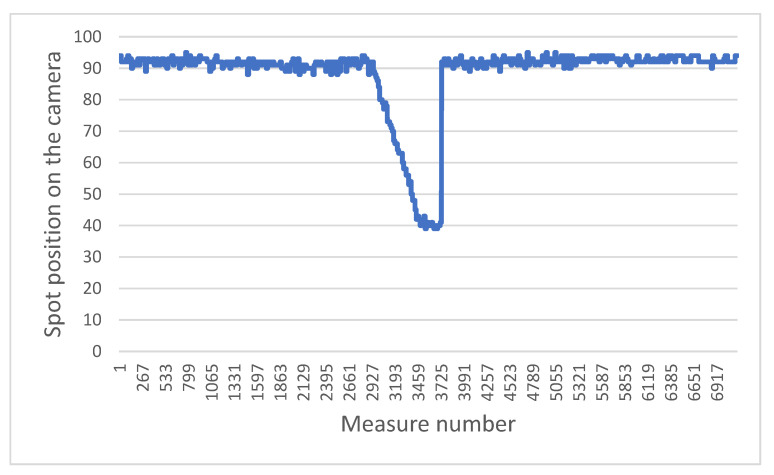
Evolution of position measured while the wheelchair moves toward a cardboard box on the ground, then exceeds it.

Figure 13 shows the evolution of the measurements when the chair approaches a cardboard box placed on the ground, and then passes it. It is then possible to split this graph into five phases. In the first one, roughly flat except for uncertainties, the measurements are taken from the wheelchair when it rolls along the flat ground. In the second, a gradual decrease in values corresponds to the moment when the chair meets the cardboard, and the laser beams start to rise on it as the chair moves forward. Then, in the third phase, the points are found on the upper part of the box, which is therefore flat, but closer than the flat ground (therefore still constituting an uncertainty). Finally, the beams go beyond the cardboard box and fall back to the flat ground (phase four). This is very visible thanks to the clear break at the level of approximately point 3730. Then the beams advance again on the flat ground (phase five).

According to the height difference detection algorithm presented above, the moment of the break (point 3730) signifying the return to flat ground represents a very strong derivative. Therefore, it can potentially be considered by our system to be a dangerous height difference. This is not the case, as there is simply no presence of a drop here. To avoid this, we record the value of the flat ground every time the system is started. In addition, only strong positive derivatives whose values of the points are higher than the standard values of the flat ground will be considered dangerous. That is to say, only those points at an altitude lower than flat ground. In this way, it is not only possible to avoid the false positives caused by this kind of situation, it is also possible to detect “positive” obstacles, that is to say, obstacles protruding from the ground.

**Figure 14:**
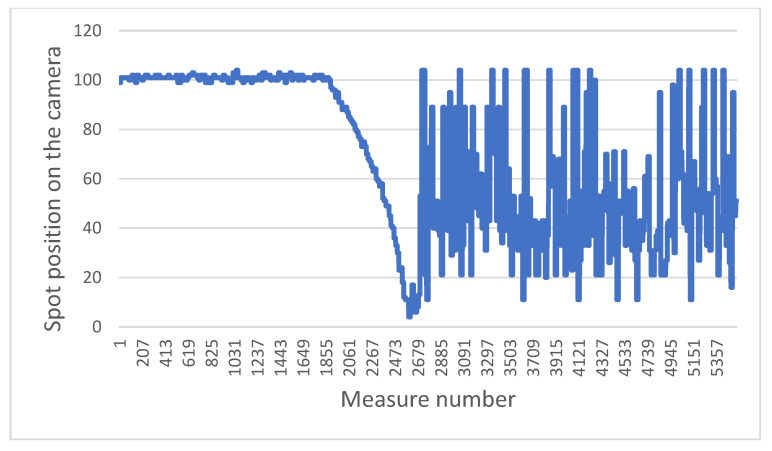
Evolution of position measured while the wheelchair moves toward a wall until the signal is lost.

Finally, Figure 14 illustrates the set of measurements when the chair approaches a wall until coming into contact with it. This case is typical of a situation where an obstacle is too close to the sensor and no beam returns to the camera. Our algorithm is therefore unable to identify a backscattered laser spot and therefore returns random values corresponding to the most sensitive areas of our camera, modulated according to ambient noise. The same thing happens when the beams fall over a precipice and the ground is therefore too far away to be detected. No beam returns to the camera, and a chain of random values is returned.

#### 3.2.3. Treatment of Random Values

First of all, a sequence of random values is quite easy to identify using our algorithm. It suffices to measure the standard deviation between the six points used for the detection of height difference. If this standard deviation exceeds a threshold previously set after a series of dedicated tests, then two possible situations emerge. Either an obstacle is very close (such as a wall or an object protruding from the ground), or there is an extremely high difference in level in front of the wheelchair (such as the bottom of a staircase or a deep ditch in the path).

So how to differentiate between the two situations? Well, we can rely on the other sensors present on the wheelchair, namely those intended for the anti-collision function. This consists of a set of infrared and ultrasonic sensors which point horizontally all around the chair. In the case of a wall or a very close object, the anti-collision sensors will also detect it. Conversely, in the case of a staircase or a ditch, nothing will be visible to these sensors. Thus, the algorithm has two different behaviors depending on the results obtained by the different sensors. If the fall arrest sensors measure a random set of values and the collision avoidance sensors measure nothing close, this means that there is a steep drop in front of the chair. Then, all commands directing the wheelchair in the direction of the height difference will be prohibited. However, if the fall arrest sensors measure a set of random values and the collision avoidance sensors observe a very close obstacle, the wheelchair will be allowed to move forward at an extremely low speed. In this way, the user will always be able to move around in this narrow environment without risking strong collisions with their environment.

#### 3.2.4. Video Illustrations

We give below some links to videos that illustrate several situations. The videos are also available on the publisher’s website.

The first video (https://www.youtube.com/watch?v=u8DGt_kkERA (accessed on 20 September 2021) shows a non-disabled user moving towards a staircase or a sidewalk and verifying that the system stops the wheelchair only in that direction. All this is performed indoors and outdoors, including in full sun.

The second video (https://www.youtube.com/watch?v=RdBgFwNnrQ0 (accessed on 20 September 2021) features a member of our research team, who is blind from birth, in different complex situations. Unlike the first video, this one also includes demonstrations of the anti-collision functions of our system. Which will be the subject of another article, as previously mentioned.

## 4. Discussion

### 4.1. Relevance of the Developed Sensor

Having finished developing our sensor, and having mounted it on the power wheelchair and tested it under different conditions, it is now time to try to assess its suitability in comparison to the sensors available on the market and generally used in similar projects.

Before doing so, it is important to remember that a sensor, whatever its nature, is the result of a large number of conditions. In the most important elements of these compromises we find: the precision and the resolution of the device, i.e., the minimum step of the measurement, in all directions of space; its field of detection (more or less wide); the cadence with which it takes its measurements; its robustness (functional in full sun, at night, in the rain, with what types of obstacles, etc.; its size; the energy it consumes; its cost (which essentially depends on the time spent developing it and on the materials that compose it).

Usually, setting one of its criteria will automatically affect one or more of the others.

Now let us look at these criteria in our scope of application, namely the protection of the wheels of an electric wheelchair to prevent it from falling from sidewalks or stairs.

- Its precision, namely 1.5 cm on average in the distance ranges that interest us, is less than or equal to what is generally available on the market (ultrasound sonars, infrared sensors or laser LiDARs, which generally possess a precision equal or inferior to 1 cm). However, 1.5 cm is more than sufficient for determining the possible dangerousness of a drop.
- Its detection field is a bit special and is difficult to compare, since it consists of a point detection, multiplied by the number of points distributed on a line on the ground in front of the wheelchair wheels. This allows better spatial resolution than ultrasonic sonar type sensors or infrared sensors, because these emit a large cone in front of them, not making it possible to evaluate the flatness of the ground.
  In our opinion our solution is more suited to our context than rotating laser LiDARs, which have difficulty targeting a region on the ground around the wheelchair wheels, and the majority of the measurements of which would be irrelevant because they are in the air due to the rotating nature of this type of device. It would be possible to achieve a detection field similar to ours, or even better, by associating several punctual LiDARs. However, that would be nonsense in terms of congestion and energy consumption.
  It is necessary to have something that looks like a series of points on the ground, like the operation of the Kinect; however, this type of technology cannot be employed outdoors because of sunlight.
- The measurement rate (cadence) is roughly on the same order of magnitude as most sensors on the market. This is not a big issue here, as the wheelchair equipped with our system moves at a maximum speed of 3 km/h. Considering a frequency of measurement of 30 Hz per point on the ground, it is calculated that the chair can at best travel 2.7 cm between each measurement. This is more than sufficient given the arrangement of the laser beam and the necessary braking distances (see: Section 2.3.5. Arrangement).
- Its robustness of detection makes it possible to correctly detect height differences in all indoor and outdoor conditions, which is only true for high-end LiDARs on the market (Velodyne, Ouster or Sick, for example).Its mechanical robustness, that is to say, its solidity and its capacity to resist over time, is still too weak to be compared to what is available on the market. This is undoubtedly the difference between an experimental system and an industrial system.
- Its dimensions (3.2 cm × 3 cm × 2.2 cm) classify it as a small sensor, easily integrated into the volume of the chair, without anything protruding or obstructing navigation. Unlike punctual or rotating LiDAR sensors, which are typically larger. Even more so if they are robust to sunlight.
- Its consumption (0.5 Watts) is greater than ultrasonic sonars and infrared sensors, but way lower than the LiDARs available on the market, where even the most economical in terms of energy consumes at least 15 W.
- Its price is difficult to evaluate at this time, since this is not an industrial product. Although we have an idea of its current material cost (around EUR 150), it is impossible to assess its real cost, since it is based on several years of research. By way of comparison, an entry-level functional exterior LiDAR is around EUR 2000.

To conclude this section, although there are systems capable of providing the same functions as our sensor, especially in the LiDAR family, limitations still persist. The size and consumption of commercial LiDARs that are roughly adapted to our environment of use are not compatible with electric wheelchairs in daily use.

### 4.2. Wearable Device

An electric wheelchair is a daily commuting tool for its user, like a pair of shoes that keeps them going. In the same way that it is inconceivable for a non-disabled person to go out without a pair of shoes, so it is for a wheelchair user without their wheelchair.

This is true to such an extent that many wheelchair users consider it an integral part of their body [25]. In addition, it is not simply a question of a feeling, since several studies have even looked at the way in which the brain adapts to the wheelchair and considers it to be part of the human body [26], whether with respect to feeling or to the proprioception of the user [27].

This is why wheelchairs, manual as well as electric, are considered “wearable devices”. A major stake of our project, in its entirety, is to retain this “wearable” aspect of the wheelchair. This is why it is essential that our system be able to connect almost invisibly to all powered wheelchairs on the market. It should be almost physically invisible, so as not to increase the volume of the wheelchair and disrupt navigation, but it should also have modest electronic requirements, as too much consumption would disrupt the daily journeys of the user, increasing the frequency of recharging.

Our sensors meet these constraints in terms of size, weight and consumption.

Therefore, their use could be extended to other applications. For example, on a similar theme, a variant of these sensors is being developed by our team for electronic white canes [28], where it will be used to anticipate the presence of drops in order to transmit information to the user. Here again, the portable peculiarity of the sensor is essential since it is directly attached to the user’s white cane, influencing its weight and requiring low power consumption, such that the latter can be powered by an electric battery, in order to preserve its wearable characteristics.

### 4.3. Perspectives

Although our sensor serves as a good proof of concept with respect to its operation for this type of application. It would be advantageous to improve several aspects:

#### 4.3.1. Multibeam

Our sensor currently works with three beams. This allows us to cover a reasonable line in front of the electric wheelchair, permitting a safe navigation for both frontal and lateral directions. However, it would be interesting to increase the number of beams and to modify their projection on the ground. Rather than a line, it would be relevant to project the laser points in a semi-circle in front of the wheelchair wheels, in order to increase the density of the detection mesh and improve its spatial resolution. This would allow us to refine our algorithms and allow safer and smoother browsing.

#### 4.3.2. Mechanical Robustness

As mentioned above the alignment between our lens and the camera of our sensors varies constantly by a few micrometers, or even millimeters in the worst cases. This is mainly due to constant vibrations and occasional shocks. Our algorithm, in its initialization phase, systematically auto-calibrates the system in order to smooth out such problems. However, this is a limitation. Therefore, it is important to improve the mechanical robustness of our sensor and its resistance with respect to time.

### 4.4. User Cases Studies

As mentioned in the introduction, it is complicated to define a precise specific population likely to use our system, quite simply because the population requiring electric wheelchairs to be able to move around, but which does not have access to them for safety reasons is diverse and varied. Indeed, the impairments leading to such a situation are often of neuromotor origin, with a cognitive and/or visual component. While many can be identified as belonging to the cerebral palsy family, this is not exclusively the case, and above all, this pathology is expressed in a different way for each individual. Even for a sole individual, it is possible to observe differences in their abilities within the same day, depending on fatigue and the emotional state of the person.

For this reason, we prefer to focus on the abilities and limitations of each person, regardless of the root causes of their condition.

Thereby we carried out a certain number of tests with different user profiles. Below we describe the various tests performed, in the most objective way possible, while attempting to describe the capacities and limitations of the users as well as their experience of the system.

There are six separate cases.

The first was a young man affected by a rare congenital disease, causing in him a loss of sensitivity of the lower and upper limbs as well as a quasi-blindness. His loss of sensitivity prevented him from moving around using his legs or from standing for a long time. Additionally, although he had no hand sensitivity, he was able to exert some control over the joystick of a power wheelchair, but with little precision. His vision had deteriorated over time and at the time of the tests had an acuity of less than 1/50 in a 5% field. His cognition was excellent, and despite his impairments, he was taking lessons in the final year of high school. He had also been able to pilot his chair alone when he had sufficient vision, although this was no longer possible. Several tests were carried out in his school, which showed a great capacity for adaptation and the implementation of strategies allowing him to move again independently in a known place. He has also very recently spent 3 weeks of vacation at a campsite that he knew very well (under his parents’ supervision) with his wheelchair equipped with our system. He is our first daily user, and the feedback has been very positive.

The second was a young girl, hosted in a specialized center, with cerebral palsy, who presented a narrow visual field, a weak capacity of concentration and a psychomotor control that did not allow her to perform fine movements. She was able to steer a wheelchair on her own but with a certain number of collisions, due to her inability to follow an established trajectory. The results were satisfactory, since she was quickly able to take a walk outside without anyone intervening on the controls of the chair, and then to stroll in the corridors of her center at recreation time (so with a lot of other children around) without making a single collision.

The third was a child with cerebral palsy, mainly with cognitive impairment that did not allow him to move around on his own in an electric wheelchair. He was able to move smoothly in the courtyard of the center where he was staying and put in place strategies to try to get out of the center in question. Although autonomous navigation was made possible for him, he remained excluded from access to an autonomous travel solution, due to his cognitive impairment. Indeed, the ability to move is not enough, you must also be responsible for your travels. The danger does not necessarily come from collisions and falls, but also from situations in which the user may find themselves (on the roadway, in unauthorized premises, etc.), and it is necessary to be able to understand and respect the rules of the environment in which the person finds themselves.

The fourth was a man in his forties, paraplegic with weak upper limbs, completely blind, with good hand control on his wheelchair. He was able to operate his chair on his own before he lost his sight completely. Since then, he has been living in a specialized center, which he knows perfectly well and in which he operates using his electric wheelchair and a long cane that he sweeps along the wall to guide the chair. After a few hours of using our system, he perceived the considerable work required to be able to use it fully. Although after a certain period of adaptation he managed, outdoors, to follow a path avoiding two people voluntarily placed on it, he wondered if he had the necessary motivation to learn such a system. The problem is the spatial representation he needs to develop to follow trajectories that are not guided by walls.

The fifth was a young man with cerebral palsy, with total blindness, severe cognitive impairment and with great difficulty in taking initiative. At the time of our meeting, he was in a manual wheelchair pushed by a third person. Unfortunately, these tests were inconclusive. The user was very uncomfortable driving an electric wheelchair he was not used to. He did not seem very receptive to the instructions of the tests we were trying to perform and remained still for the most part.

The sixth and last user met was a young man with mild cerebral palsy, leading to moderate motor control, and a loss of the visual field causing difficulty in assessing the depth of relief on the ground. Very good cognitively, he was able to pilot his wheelchair alone, without hindrance, in known places and without relief on the ground. Our system limiting the wheelchair to 3 km/h was too frustrating for him, as he is usually able to drive the wheelchair at full speed in a known environment. The best freedom/security compromise for him is to continue to use his wheelchair freely in places he knows and to avoid unknown and potentially dangerous places, or accompanied by a sighted person.

Below, Table 2 sums up the six cases presented.

## 5. Conclusions

We investigated the challenges related to providing access to autonomous transport solutions for people with disabilities or dependent elderly people. After describing typical populations for whom there is currently no solution, as well as their limitations, we endeavored to set out all the conditions that would have to be met in order to be able to offer them an adequate solution.

In this report, we were particularly interested in fall arrest solutions that could be installed in electric wheelchairs for this type of population. In particular, we addressed this by presenting a new sensor, especially designed to check the flatness of the ground and detect the presence of dangerous unevenness or obstacles on the ground. Particular attention was paid to the description of its sensors, and to the conditions that need to be met, in particular with respect to the resolution necessary for this type of situation and its robustness to any environment, indoors as well as outdoors.

A large amount of work still has to be done, but our initial feedback from concrete users leaves us confident regarding the future of this project.

## Figures and Tables

**Table 1:** Breaking distance according to the speed of the electric wheelchair.

Measured Speed (km/h)	Braking Distance (cm)
2.1	34
3.0	66.5
6.4	122.2
8.8	184.3
10.5	217.2

**Table 2:** User cases studies summary.

User’s Profile	Assessment of Tests	Observations
Post-adolescent man. Congenital disease resulting in loss of sensitivity of the limbs and near blindness.	Very satisfactory	Very good adaptation to the system, is beginning to concretely use it daily, in an autonomous way, under supervision.
Pre-teenage girl with cerebral palsy. Rough hand control.	Satisfactory	Good progress and unhindered navigation. The system may be useful for this user.
Child with cerebral palsy and cognitive impairment. Unaware of the danger.	Moderate to satisfactory	Function correctly fulfilled by our system, but the unconsciousness of the danger and the established rules do not allow a secure employment.
Adult in his forties. Blind and paraplegic.	Moderate	Function correctly fulfilled by our system but the spatial representation it implies and the need to adapt to it is difficult for some, especially over a certain age.
Young man with cerebral palsy, total blindness, severely cognitive impaired, great difficulty in taking initiative.	Unsatisfactory	The system is useless in this situation, since the user does not initiate any movement.
Teenage boy with mild cerebral palsy, sufficient hand control, difficulty perceiving the relief on the ground.	Unsatisfactory	Our system is too secure and frustrating for its capabilities.
